# Single‐cell transcriptomics reveals pathogen interactions and T cell reprogramming in HIV and *Mycobacterium tuberculosis* co‐infection

**DOI:** 10.3389/fimmu.2025.1680538

**Published:** 2025-11-28

**Authors:** Zihui Zhao, Suyue Huang, Wei Huang, Wei Song, Li Liu, Jun Chen, Renfang Zhang, Yinzhong Shen

**Affiliations:** 1 Shanghai Public Health Clinical Center, Fudan University, Shanghai, China; 2 Shanghai Institute of Infectious Disease and Biosecurity, Fudan University, Shanghai, China; 3 Zhongshan Hospital, Fudan University, Shanghai, China

**Keywords:** HIV-Mtb co-infection, single-cell transcriptomics, T cell exhaustion, Th1/Th17 imbalance, TNF signaling

## Abstract

**Background:**

Human immunodeficiency virus (HIV) and *Mycobacterium tuberculosis* (Mtb) co-infection remains a major cause of mortality in AIDS patients, yet the mechanisms of pathogen interplay and host immune remodeling remain poorly understood.

**Methods:**

To capture early untreated states, we applied single-cell RNA sequencing (scRNA-seq) to peripheral blood mononuclear cells from healthy controls, and from participants newly diagnosed with HIV mono-infection or HIV-Mtb co-infection, before therapy initiation. Integration guided by a Directed Acyclic Graph (DAG) inferred a pseudo-temporal trajectory from health to HIV infection to co-infection.

**Results:**

Along this continuum, TNF-α and TGF-β signaling progressively declined in CD8^+^ T cells and monocytes. Th1 cells emerged as the dominant anti-*tuberculosis* effectors, whereas Th17 cells exhibited transcriptional exhaustion and ribosomal stress signatures consistent with a non-responsive state. Cell communication analysis revealed fewer overall interactions but increased signaling strength within pathways during co-infection. Notably, we observed a transition in T cell from MHC class II to class I, a shift that was most pronounced in the CD4^+^ effector memory subset. These rewired interactions featured selective upregulation of inhibitory checkpoint molecules (PGE2–PTGES3–PTGER2/4, PPIA-BSG, PECAM1) and loss of stimulatory signals (CD6-ALCAM, CLEC2B/C/D-KLRB1).

**Discussion:**

Our study provides a single-cell roadmap of HIV-Mtb co-infection and identifies Th1/Th17 imbalance and MHC-I-biased T-cell signaling reconfiguration as candidate targets for restoring immune homeostasis.

## 1 Introduction

Human immunodeficiency virus (HIV) infection causes progressive immune destruction, leading to severe acquired immunodeficiency syndrome (AIDS). Co-infection with *Mycobacterium tuberculosis* (Mtb) continues to contribute significantly to global mortality in this vulnerable population. According to the *WHO Global tuberculosis report 2024*, approximately 10.8 million *tuberculosis* (TB) cases were reported in 2023, with 6.1% occurring in HIV-positive individuals in the same year, and TB-associated deaths reached 1.25 million, including 161,000 among HIV-positive patients (1). Moreover, data released by the Joint United Nations Programme on HIV and AIDS (UNAIDS) in 2024 indicated that around 630,000 people died from HIV-related diseases in 2023, underscoring Mtb’s persistent role in AIDS mortality (2). Regional studies in China further illustrate this public health challenge. A retrospective study in Guangxi (2011–2019) revealed that TB accounted for 35.3% of opportunistic infections in hospitalized AIDS patients (3). These findings underscore the urgent need for targeted interventions to address the HIV-Mtb syndemic.

HIV-Mtb co-infection is biologically distinct from either mono-infection and is marked by bidirectional pathogenic interactions. HIV infection increases the risk of active and disseminated tuberculosis, whereas Mtb infection promotes HIV replication and dissemination, amplifying immune dysregulation and inflammatory responses (4, 5). Mechanistically, this synergistic pathogenesis arises from compounded immune destruction and microenvironmental dysregulation. Mtb facilitates HIV by inducing inflammatory factors such as nuclear factor of activated T cells 5 (NFAT5) (5, 6), while upregulating key co-receptors (CCR5 and CXCR4) that facilitate viral entry into host cells (5). Concurrently, HIV compromises macrophages’ ability to eliminate Mtb (7–9), disrupts their antioxidant capacity, and facilitates Mtb growth (10). It also induces dysfunction in natural killer (NK) cells and depletes Mtb-specific CD4^+^ T cell populations, further contributing to TB progression (11–13). Nevertheless, most studies have relied on pairwise contrasts between mono- and co-infection, providing limited insight into the dynamic host–pathogen interplay.

**Figure 1:**
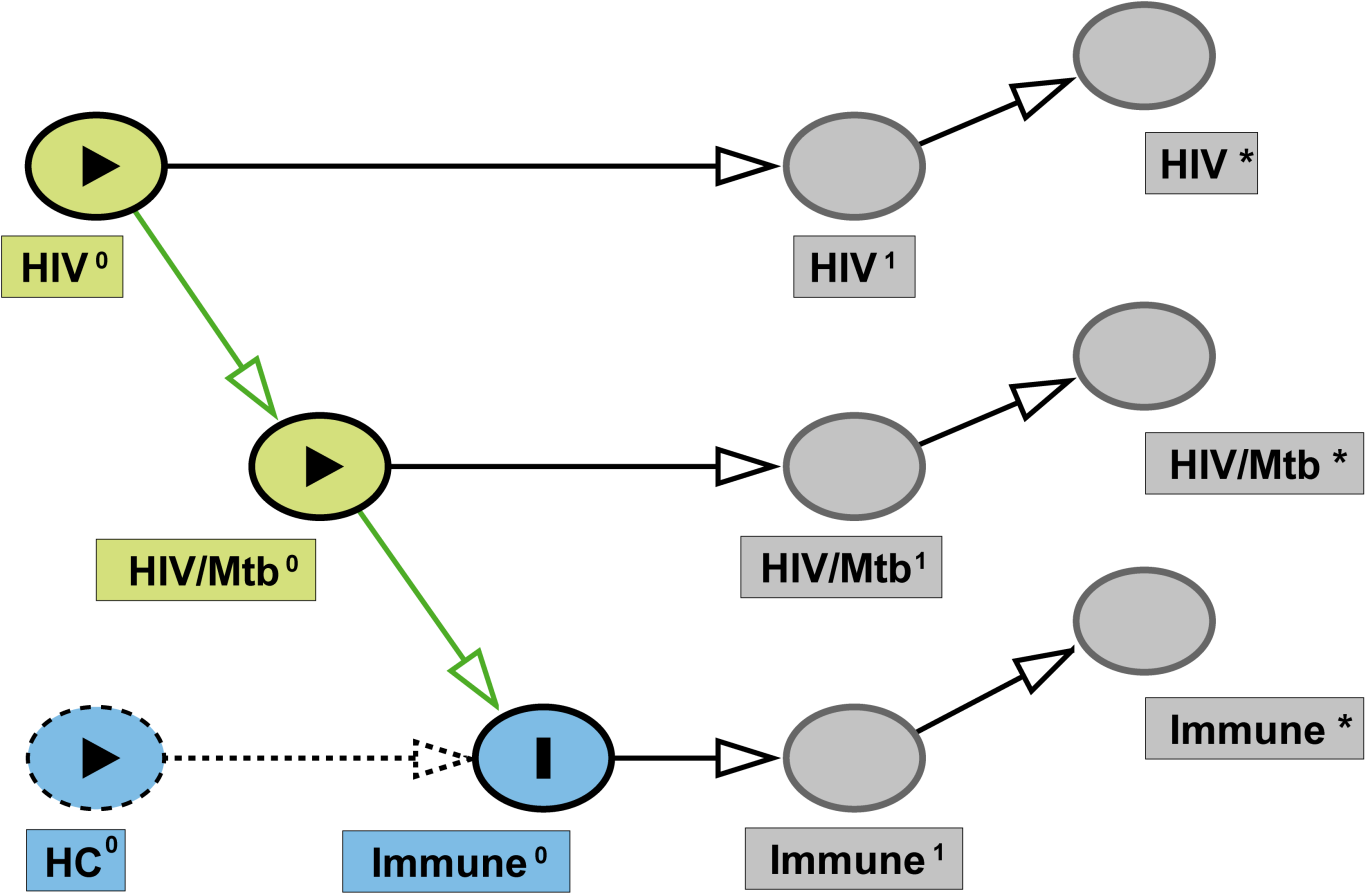
Directed acyclic graph (DAG) of study design. Latent baseline exposures— HIV^0^ and HIV/Mtb^0^ —are depicted as green nodes, and the study outcome (immune status) is represented by Immune^0^ (blue). Dashed blue node indicates the HC group, serving as the reference category rather than an active causal variable. Superscript “1” denotes the true underlying state at the time of sampling (HIV¹, HIV/Mtb¹, Immune¹), while superscript “*” indicates the corresponding observed measurements in this study (HIV^*^, HIV/Mtb^*^, Immune^*^). Arrows represent the assumed causal relationships from baseline exposures to measured outcomes.

In this study, we performed single-cell RNA sequencing (scRNA-seq) on peripheral blood mononuclear cells (PBMCs) in a cross-sectional design. The design was guided by Directed Acyclic Graph (DAG) (**Figure 1**), framing healthy controls (HC), HIV-mono infected individuals, and HIV-Mtb co-infected individuals as a pseudo-longitudinal continuum of disease. We sought to investigate pathogen interactions and delineate the evolving immune landscape throughout disease progression.

## 2 Materials and methods

### 2.1 Study approval

Ethical approval for this study was obtained from the Ethics Committee of Shanghai Public Health Clinical Center (approval number: 2022-S012-02). The research was conducted in full compliance with the Declaration of Helsinki and relevant ethical guidelines, with all samples used solely for the purposes outlined in the study protocol. Personal data were maintained in strict confidence. Written informed consent was obtained from the individuals for the publication of any potentially identifiable images or data included in this article.

### 2.2 Analytical scope and sex representation

**Figure 2:**
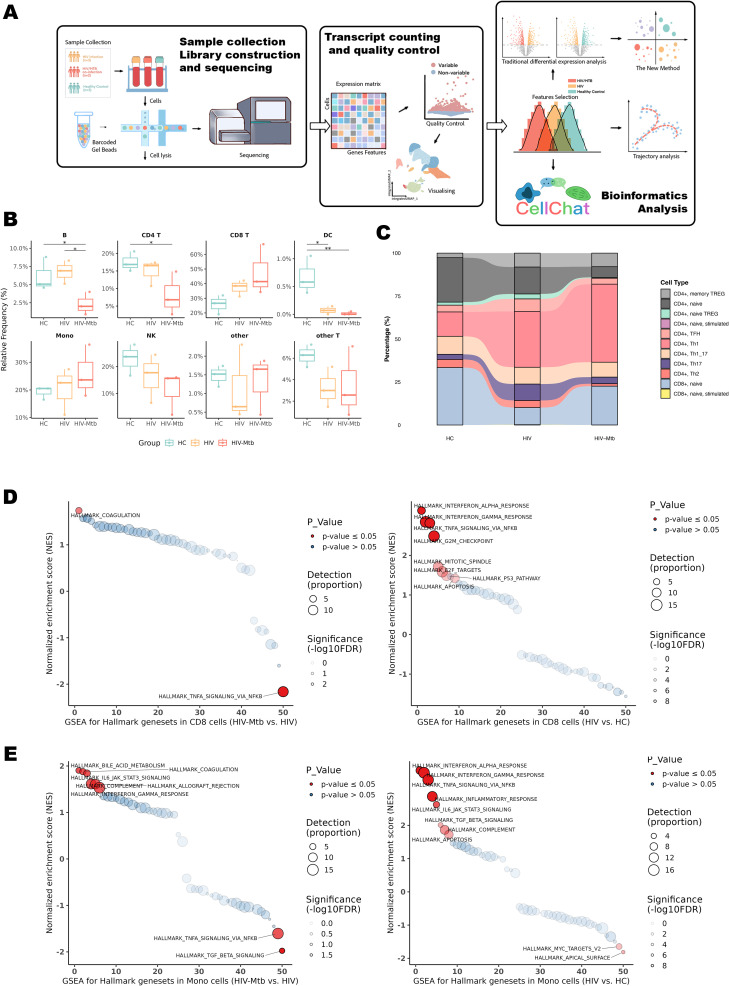
Workflow of the present study and single-cell sequencing subpopulation analysis. **(A)** Schematic flowchart of the sample-processing workflow. **(B)** Analysis of major cell subpopulations annotated by Azimuth Level 1. * indicates P < 0.05 and ** indicates *P* < 0.01 for the respective groups. **(C)** Stream plot depicting T-cell subpopulation annotation generated using the DatabaseImmuneCellExpressionData from SingleR. Statistical significance for this plot is provided in **Supplementary Table S1**. **(D)** GSEA analysis of 50 Hallmark pathways in CD8^+^ T cells across three groups; left: HIV-Mtb group vs. HIV group; right: HIV group vs. HC group. Pathways marked in red indicate those with an FDR < 0.05. Detection (Proportion): Circle size represents the number of enriched genes scaled by a factor of 10 (larger circles indicate a greater number of genes), and the darkness of the circle outline corresponds to log10(FDR), with darker colors indicating lower FDR values. **(E)** GSEA analysis of 50 Hallmark pathways in monocytes across three groups; left: HIV-Mtb group vs. HIV group; right: HIV group vs. HC group.

This study focused on early, treatment-naïve immune states across the disease course (HC → HIV-mono → HIV-Mtb). In the routine clinical population of HIV-Mtb co-infected patients managed at our center during the study period (**Supplementary Figure S1**), the sex distribution was male:female = 196:30 (86.7% were men). This pattern is consistent with regional epidemiology reported in the *UNAIDS 2025 Global AIDS Update* (page 66, Figure 2), where approximately 74% of new HIV infections in the Asia–Pacific region occur in men (14). From this ongoing clinical population, three newly diagnosed, treatment-naïve adults who provided informed consent were enrolled for the present exploratory single-cell sub-study. All participants were men. Given the exclusively male enrollment and the small sample size, analyses were restricted to men to avoid sex-related heterogeneity. Accordingly, the findings pertain to treatment-naïve men with HIV-Mtb co-infection, who constitute the majority of new HIV infections in the Asia–Pacific region. Future cohort expansions will include sufficient numbers of women to allow sex-stratified analyses.

### 2.3 Study participants

Written informed consent was obtained from all participants prior to enrollment. The detailed inclusion criteria for patients were as follows: (i) newly diagnosed with HIV infection/AIDS; (ii) newly diagnosed with TB. The exclusion criteria were: (i) prior use of antiretroviral therapy (ART) or anti-tuberculosis drugs; (ii) detection of drug resistance in HIV susceptibility tests; (iii) severe liver and/or kidney dysfunction; (iv) presence of underlying conditions such as pancreatitis or malignant tumors. Based on these criteria, a total of 6 patients were enrolled in the study, including 3 HIV/AIDS patients with Mtb co-infection (none of whom had received either anti-tuberculosis or antiviral treatment) and 3 early-diagnosed HIV/AIDS patients (none of whom had received antiretroviral treatment) and their peripheral blood (10 mL) was collected. An *a priori* directed acyclic graph (DAG; **Figure 1**) was deployed to represent hypothesized links between HIV-mono infection, HIV-Mtb co-infection, potential confounders, and immune outcomes, thereby informing the selection of comparison groups and covariates. In view of the cross-sectional design, the DAG was employed as a conceptual and analytical framework to assist interpretation, without asserting causal relationships. The detailed information of the patient can be found in **Table 1**. All individuals were treatment-naïve and had no prior TB history. Samples were collected within two weeks of confirmed HIV diagnosis. In our local cohort, both HIV and HIV-Mtb co-infected patients were predominantly young and middle-aged men. Accordingly, healthy male controls aged 25 years without underlying diseases were enrolled.

**Table 1:** Clinical characteristics of HIV/AIDS and co-infection with Mtb patients.

Parameters	HIV/Mtb1	HIV/Mtb2	HIV/Mtb3	HIV1	HIV2	HIV3
Age (yr)	34	38	67	36	31	73
Gender	Male	Male	Male	Male	Male	Male
Viral load (copies/mL)	67300	208000	1230000	1100000	58700	55700
Co-infection with other pathogens	No	*Epstein–Barr Virus*	No	No	No	No
Date of first positive HIV test	2 weeks	0 week	0 week	0 week	0 week	0 week
T cells count in peripheral blood (/μL)						
CD4 T cell	141.79	91.24	15.71	159.72	326.65	203.95
CD3 T cell	1043.41	1147.73	151.65	966.32	1021.03	778.24
CD8 T cell	871.59	1033.8	136.68	675.54	614.96	514.13
CD45 T cell	1512.98	1199.86	203.88	1449.27	1668.07	996.69
Liver function						
ALT (U/L)	95	58	10	10.7	28.7	22
AST (U/L)	49	54	20	17.2	17.8	21.8
TBIL (μmol/L)	14	16.6	6.8	8.17	25.9	11.87
ALB (g/L)	31.8	23.5	29.5	46.31	50.63	51.91
GGT (U/L)	379	661	110	14	25	41
Renal function						
β2-MG (μg/L)	11146.51	42533.37	18737.04	11152.02	116.23	78.75
MA (mg/L)	94.3	>300	3.4	276.3	0.1	>330
UCr (μmol/L)	28214.92	3916.06	8075.74	24525.91	3813.36	17462.2
SCr (μmol/L)	120.4	82.6	71	65.5	81	105.4
Tuberculosis-Specific Pathogen Examination Results	Xpert Mtb/RIF	Culture Positive	Xpert Mtb/RIF	–	–	–

Mtb, mycobacterium tuberculosis; HIV, human immunodeficiency virus; ALT, alanine aminotransferase; AST, aspartate aminotransferase; TBIL, serum total bilirubin; ALB, serum albumin; GGT, L-γ-glutamyl transferase; β2-MG, urinary beta-2 microglobulin; MA, urinary microalbumin; Ucr, urinary creatinine; SCr, serum creatinine; Xpert Mtb/RIF, Xpert mycobacterium tuberculosis/rifampicin assay.

### 2.4 Blood sample processing

Reagent and consumable information were provided in **Table 2**. The samples were transported on dry ice as quickly as possible and centrifuged (2000 RPM, 10 min, RT), and plasma was aspirated in a biosafety cabinet, aliquoted, and PBS was added to blood tubes (total ~10 mL), gently mixed, then layered over 4 mL lymphocyte separation medium (LSM) in a 15 mL tube. After centrifugation (1200 RPM, 30 min, RT), the middle cloudy layer was transferred to a new tube, washed with PBS and centrifuged (1200 RPM, 10 min). The pellet was lysed with RBC lysis buffer (1:5 ratio, 1000 RPM, 5 min), washed twice with PBS, and resuspended in PBS containing 10% FBS. Cell viability (>95%) was assessed using Trypan Blue exclusion with an automated cell counter.

**Table 2:** Key resources table.

	Source	Identifier
Software and algorithms		
MobiVision Version 3.1	MobiDrop (Zhejiang) Co., Ltd.	https://www.mobidrop.com/bioinformatics-analysis-software/mobivision-news/software-download
Seurat Version 5.1.0	Yuhan Hao et al., 2023	https://github.com/satijalab/seurat
SCP Version 0.5.1	Zhang Hao, 2023	https://github.com/zhanghao-njmu/SCP
clusterProfiler Version 4.14.6	Tianzhi Wu et al., 2021	https://yulab-smu.top/biomedical-knowledge-mining-book/
fgsea Version 1.32.0	Korotkevich G et al., 2019	https://bioconductor.org/packages/release/bioc/html/fgsea.html
ggplot2 Version 3.5.1	Hadley Wickham, 2016	https://cran.r-project.org/package=ggplot2
ggalluvial Version 0.12.5	Brunson JC et al., 2020	https://cran.r-project.org/package=ggalluvial
ggpubr Version 0.6.0	Alboukadel K et al., 2023	https://cran.r-project.org/package=ggpubr
ggbeeswarm Version 0.7.2	Erik C et al., 2023	https://cran.r-project.org/package=ggbeeswarm
EnhancedVolcano Version 1.24.0	Kevin Blighe et al., 2024	https://github.com/kevinblighe/EnhancedVolcano.
Monocle3 Version 1.3.7	Junyue Cao et al., 2019	https://cole-trapnell-lab.github.io/monocle3/
CellChat Version 2.1.2	Suoqin Jin et al., 2024	https://github.com/jinworks/CellChat
genekitr Version 1.2.8	Yunze Liu et al., 2023	https://github.com/GangLiLab/genekitr
dplyr Version 1.1.4	Wickham H et al., 2023	https://cran.r-project.org/package=dplyr
tidyr Version 1.3.1	Wickham H et al., 2024	https://cran.r-project.org/package=tidyr
SingleR Version 2.8.0	Aran D et al., 2019	https://bioconductor.org/packages/release/bioc/html/SingleR.html
Azimuth Version 0.5.0	Hao et al., 2021	https://github.com/satijalab/azimuth
celldex Version 1.16.0	Aran D et al., 2019	https://github.com/SingleR-inc/celldex
R Version 4.4.1	R Core Team	https://www.r-project.org
Critical commercial assays		
MobiCube^®^ Single-Cell 3’RNA-seq Kits	MobiDrop (Zhejiang) Co., Ltd.	PN-S050200101
Reagents and consumables		
1× PBS(0.01M, pH7.4)	Keygen Biotech	KGB5001
Lymphoprep	STEMCELL Technologies	Catalog # 18061
Red Blood Cell Lysis Buffer	Beyotime Biotechnology	C3702
Certified Foetal Bovine Serum	Biological Industries	04-001-1ACS
3ml Pasteur Pipettes, sterile	Beyotime Biotechnology	FPIP004
15 mL sterile centrifuge tube	Shanghai Yueyibio	YB0019-15
TC20 Automated Cell Counter	BIO-RAD	TC20
TC20 disposable counting slides	BIO-RAD	145-0011
Trypan blue staining solution (0.4%)	Sangon Biotech	E607338-0030

### 2.5 Library preparation and sequencing

We used MobiNova^®^-100 Single Cell System (MobiDrop (Zhejiang) Co., Ltd.) for library preparation. Quality-controlled cells were loaded onto a microfluidic chip with beads pre-coated with universal cell barcodes and UMIs, ensuring each mRNA received a unique identifier. Within these nanoliter-scale reaction chambers, mRNA was reverse transcribed into cDNA using poly(dT) primers, and the resulting library was sequenced on an Illumina NovaSeq 6000, generating reads containing the cell barcode, UMI, and transcript sequence information.

### 2.6 Post-sequencing data processing

The software and corresponding information used in this study were detailed in **Table 2**. The raw data generated from the Illumina NovaSeq were converted into fastq files using bcl2fastq2. Subsequently, MobiVision software provided by MobiDrop was employed for single-cell transcriptomic counting to generate the expression matrix. Quality control was then performed according to the standard workflow recommended by the Seurat package (15). The quality-controlled files were annotated for cell types using Azimuth (16), and the data were merged according to the study groups using the Seurat::IntegrateLayers method. For further subsetting of T cell subpopulations, CD4, CD8, and other T cells classified at the predicted.celltype.l1 level by Azimuth were initially selected, while cell types such as Eryth at level2 annotation, which might interfere, were excluded. Specific T cell subpopulations were then identified using the SingleR (17) algorithm with the DatabaseImmuneCellExpressionData as reference.

### 2.7 Inter-group comparison methods

For pairwise differential expression analysis, Seurat’s FindMarkers function was used to identify DEGs (Differentially expressed genes). Subsequently, these DEGs were visualized and functionally annotated using the clusterProfiler package (18). Comparisons among the three groups were performed using the Novel Method developed in this study. The code, usage examples, and relevant notes for this novel method were uploaded to GitHub (https://github.com/tony27786/ThreeGroupQuadDiff), providing a basis for integration with additional analysis methods or enrichment with other databases.

### 2.8 Other visualization and downstream analyses

To further characterize T cell subpopulations, we performed pseudotime trajectory analysis with the Monocle3 package (19) and cell-cell communication analysis with the CellChat package (20) alongside the CellChatDB database.

### 2.9 Statistics

Pairwise comparisons of cell-subpopulation relative frequencies between groups were performed using Welch’s t-test. Differential expression analysis of single-cell data was conducted with Seurat’s default Wilcoxon rank-sum test, and P values were adjusted by the Benjamini-Hochberg method. For GSEA, false discovery rates were likewise corrected using the Benjamini-Hochberg procedure.

## 3 Results

### 3.1 Baseline characteristics revealed T-lymphocyte dynamics in HIV mono- and HIV-Mtb co-infection

All individuals were treatment-naïve and had no prior TB history. Samples were collected within two weeks of confirmed HIV diagnosis. (**Figure 2A**, **Table 1**). Distinct immune cell composition patterns were observed across study groups (**Figure 2B**). Significant inter-group variations were observed in B cells, CD4^+^ T lymphocytes and dendritic cells (DCs) (*P* < 0.05). Notably, CD8^+^ T cell populations in the co-infection group exhibited a trend toward expansion compared to HCs (*P* = 0.0535). Quantitative assessment showed significant alterations in T cell compartment composition. Specifically, the co-infection group exhibited a marked reduction in CD4^+^ naïve T cell (P < 0.01) and Th2 cell frequencies (*P* < 0.05) relative to HCs, while Th1 cell populations significantly expanded (*P* < 0.01). Follicular helper T cells (TFH) and Th17 cells were enriched in HIV mono-infection but substantially depleted in co-infection (P < 0.05) (**Figure 2C**, **Supplementary Table S1**).

To initially assess the impact of mono-infection versus co-infection on host immunity, Gene Set Enrichment Analysis (GSEA) was conducted across key effector cell populations (**Figures 2D, E**, **Supplementary Figure S2**). In HIV vs HC, CD8^+^ T cells showed strong enrichment of IFN-α/γ and TNFα/NF-κB pathways (NES = 3.13, 2.85, 2.82; all FDR < 1×10^-8^), while monocytes displayed broad activation of IFN-α/γ, TNFα/NF-κB, IL6–JAK–STAT3, inflammatory response, and TGF-β (all FDR < 10^-6^). In HIV-Mtb vs HIV, CD8^+^ T cells exhibited downregulation of TNFα/NF-κB (NES = −2.16, FDR = 0.001) and an emergent coagulation hallmark (NES = 1.74, FDR = 0.030), whereas monocytes showed declines in TGF-β (NES = −1.98, FDR = 0.012) and TNFα/NF-κB (NES = −1.61, FDR = 0.042), along with upregulation of coagulation (NES = 1.88, FDR = 0.042) and bile-acid metabolism (NES = 1.90, FDR = 0.042). Collectively, interferon-related pathways were shared across infection states, whereas suppression of TNF/NF-κB and enrichment of coagulation and metabolic pathways distinguished HIV-Mtb co-infection from HIV mono-infection. In contrast, GSEA performed on dendritic cells (DCs) did not identify any pathways reaching the predefined significance threshold (**Supplementary Figure S3**).

### 3.2 Focused CD4^+^ T cell subsets analysis revealed distinct immune dysregulation in HIV and co-infection

**Figure 3:**
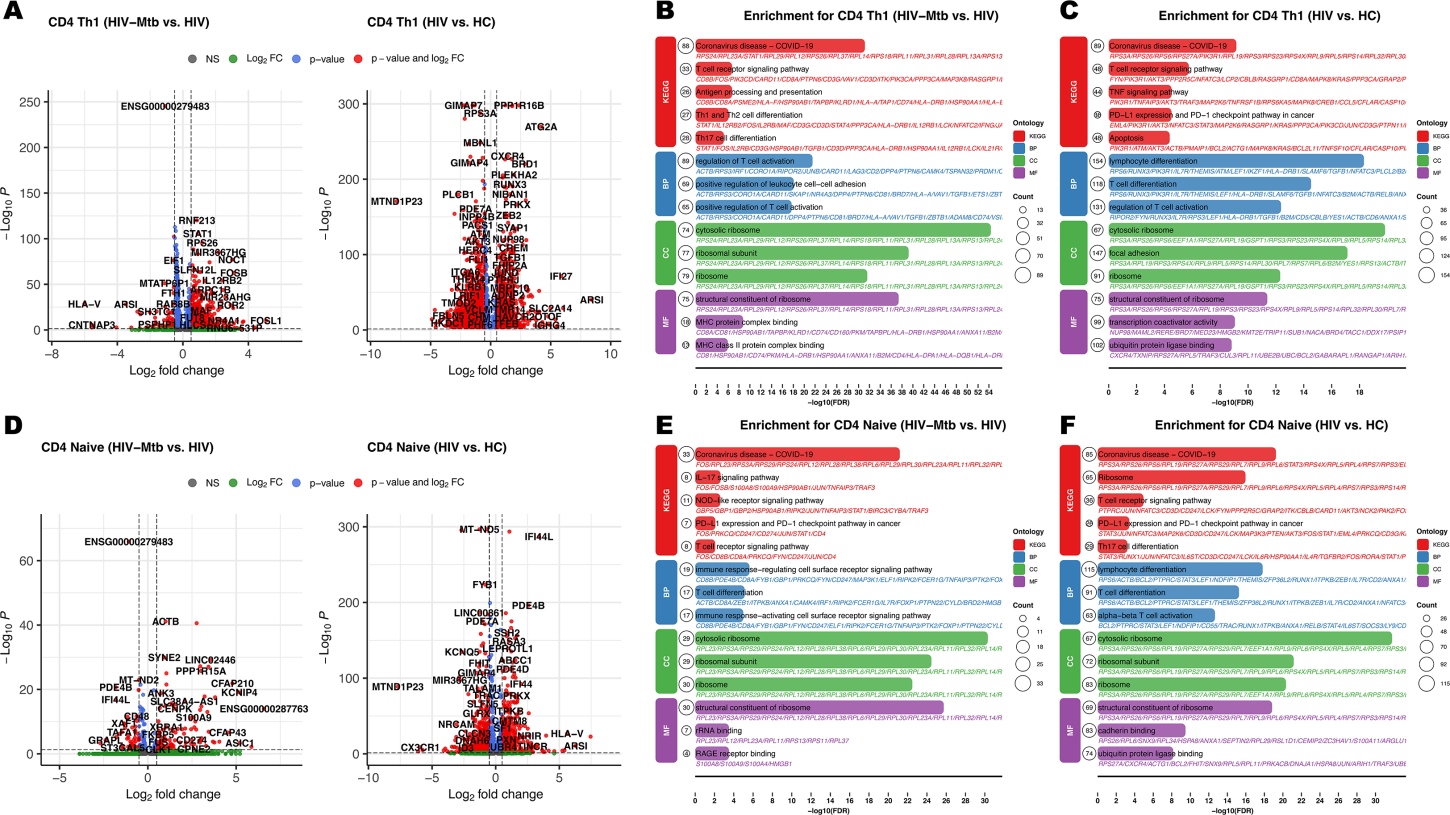
Differential expression pairwise comparison results for the CD4^+^ T cell subpopulation among three groups. **(A)** Volcano plots for pairwise comparisons in CD4^+^ Th1 subset: Left panel: HIV-Mtb vs. HIV; Right panel: HIV vs. HC. **(B)** Enrichment analysis results of KEGG (Kyoto Encyclopedia of Genes and Genomes) and GO (Gene Ontology) comparing HIV-Mtb with HIV in CD4^+^ Th1 subset; BP (Biological Process); CC (Cellular Component); MF (Molecular Function). **(C)** Enrichment analysis results of KEGG and GO comparing HIV to HC in CD4^+^ Th1 subset. **(D)** Volcano plots for pairwise comparisons in CD4^+^ Naïve subset. **(E)** Enrichment analysis results of KEGG and GO comparing HIV-Mtb with HIV in CD4^+^ Naïve subset. **(F)** Enrichment analysis results of KEGG and GO comparing HIV with HC in CD4^+^ Naïve subset.

Differential expression analysis revealed profound transcriptional alterations in four CD4^+^ T cell subsets (Naïve, Th1, Th17, TFH) during HIV-mono infection, characterized by widespread upregulation of genes linked to immune activation and inflammation, including CD38, HLA-DR, IFNG, TNF, CXCL10, and STAT1 (**Figures 3A, D**, **Supplementary Figures S4A**, **Supplementary Figures S5A**). Enrichment analyses for the HIV vs. HC comparison consistently highlighted pathways associated with T cell activation and proliferation, alongside clear evidence of immune exhaustion with enrichment of PD-1/PD-L1 (**Figures 3C, F**, **Supplementary Figures S4C**, **Supplementary Figures S5C**) (21). In addition, inflammatory pathways such as TNF signaling and Th17 differentiation were also significantly enriched (**Figures 3C,F**). The HIV-Mtb vs. HIV comparison revealed a relatively limited number of DEGs. However, enrichment analyses uncovered key shifts under Mtb co-infection (**Figures 3B, E**, **Supplementary Figure S4F**, **Supplementary Figures S5F**). There were amplified immune responses and exacerbated inflammation, especially evidenced by enrichment in pathways like TLR/NOD-like receptors and TNF signaling (**Figures 3B, E**).

### 3.3 Integrative analysis revealed sequential Th1 immune shifts, Th17 dysfunction, total memory loss, and exhaustion in HIV-Mtb co-infection

**Figure 4:**
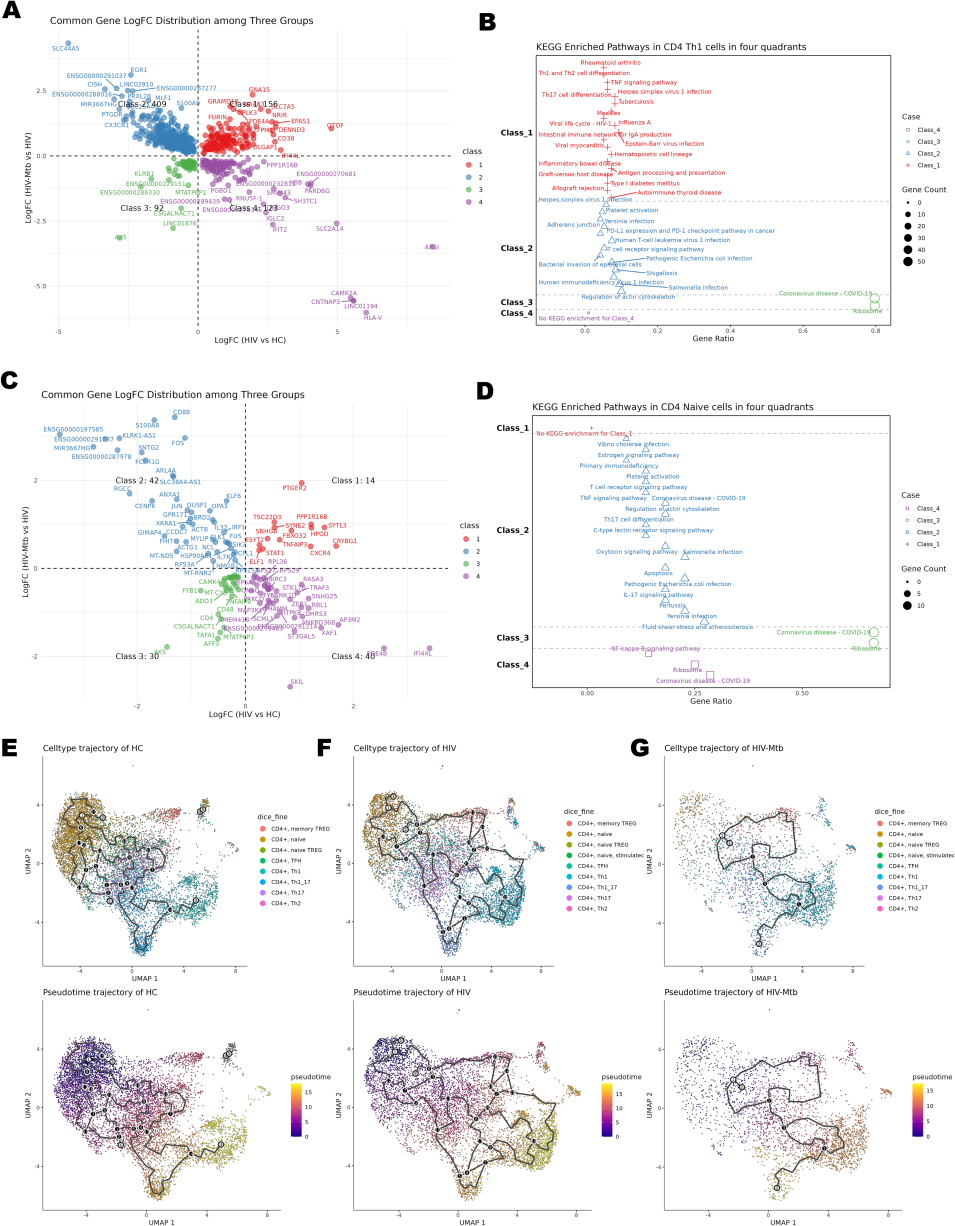
Time-series analysis of CD4^+^ T cell subsets. **(A)** Differential expression four-quadrant plot of CD4^+^ Th1 cells; the X-axis represents the comparison between the HIV group and the HC group, and the Y-axis represents the comparison between the HIV-Mtb group and the HIV group. **(B)** KEGG enrichment analysis results of the molecules corresponding to the quadrants of CD4^+^ Th1 cells. **(C)** Differential expression four-quadrant plot of CD4^+^ naïve T cells. **(D)** KEGG enrichment analysis results of the molecules corresponding to the quadrants of CD4^+^ naïve T cells. **(E)** Pseudotime Trajectory Plot for the HC Group; Top: UMAP plot with cell annotations; Bottom: UMAP plot of differentiation pseudotime, where the color gradient from purple to yellow represents differentiation time, showing the distribution of cells along the pseudotime axis. **(F)** Pseudotime Trajectory Plot for the HIV Group. **(G)** Pseudotime Trajectory Plot for the HIV-Mtb Group.

These classical analyses, however, compare only two groups at a time and do not show how the immune response evolves from HC to HIV-mono and then to co-infection. To overcome this, we mapped all DEGs onto a two-dimensional coordinate system (**Figures 4A, C**) and then performed KEGG pathway enrichment on the genes in each of the four quadrants (**Figures 4B, D**, **Supplementary Figure S6**). Molecules in the first quadrant were upregulated along both the X-axis (HIV vs. HC) and the Y-axis (HIV-Mtb vs. HIV), while molecules in other quadrants exhibited differential expression patterns corresponding to their coordinate combinations.

Th1 cells act as central drivers of inflammation in both chronic HIV and HIV-Mtb infections, yet co-infection uniquely elicits antibacterial and pro-apoptotic programs not observed in HIV alone (**Figures 4A, B**). Enrichment analysis showed significant enrichment in numerous pathways related to inflammation and immune activation in both Class1 and Class2, such as TNF signaling, Th1/Th2 cell differentiation, Th17 cell differentiation, antigen processing and presentation, and T cell receptor signaling. Additionally, infection-related pathways segregated across four quadrants. In Class1, the viral infection pathways (e.g., HSV-1, EBV) and antigen presentation (e.g., HLA-DRB1/DQB1/C, CD74, CTLA4, GZMB, PRF1) were primarily enriched. Whereas Class2 uniquely included pathways related to bacterial infections (e.g., *E. coli*, *Salmonella*) together with actin-cytoskeleton and T-cell modules (e.g., ACTB, PFN1, RAC2, PIK3CD, CD3D/CD3G, STING1, TRADD, CASP8, BCL2, HLA-F). Viral terms occurred in both classes, but bacterial pathways appeared only in Class2. By contrast, Th17 subset analysis showed enrichment solely of protein metabolism pathways under infection stress, reflecting their immune exhaustion (**Supplementary Figures S6C**, **Supplementary Figures S6D**). By contrast, Th17 subset analysis (**Supplementary Figures S6C**, **Supplementary Figures S6D**) showed enrichment solely of protein metabolism pathways under infection stress, reflecting their immune exhaustion.

CD4^+^ Naïve T cells in Class2 exhibited abnormal activation because of the enrichment of Th17/IL-17/TNF/C-type lectin pathways, together with pro-apoptotic enrichment of JUN/FOS via estrogen/oxytocin/apoptosis pathways. Class4 of the subset showed downregulated NF-κB signaling from reduced MAP3K1/BIRC3/PRKCQ (22–24) (**Figures 4C, D**).

**Figure 5:**
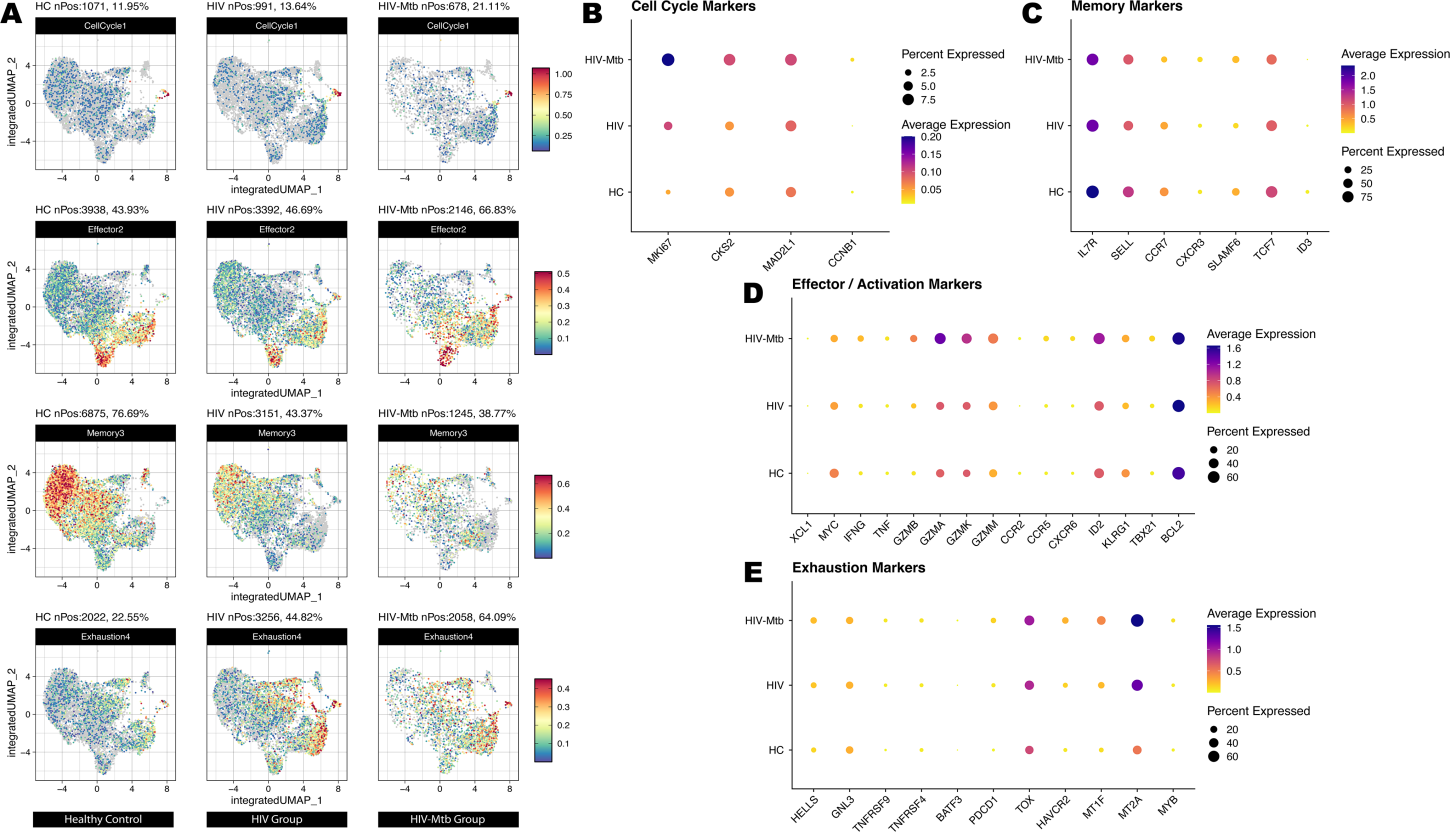
Cell states of T cell subsets on a unified UMAP embedding derived from trajectory analysis. **(A)** Module-score projection of four functional gene sets on the integrated UMAP. For each of the three sample groups (HC, HIV, HIV-Mtb), we calculated module scores for the Cell Cycle, Effector/Activation, Memory and Exhaustion gene sets and overlaid them on the same UMAP coordinate system. Rows correspond to gene‐set categories (from top to bottom: Cell Cycle, Effector/Activation, Memory, Exhaustion), and columns correspond to sample groups. Color intensity reflects the relative module‐score magnitude in each cell. **(B)** DotPlot of canonical cell‐cycle genes across HC, HIV and HIV-Mtb. Dot size indicates the percentage of cells expressing each gene within a group, and color intensity represents average expression level. **(C)** DotPlot of memory-associated genes across the three groups. **(D)** DotPlot of effector and activation-related genes. **(E)** DotPlot of exhaustion‐associated genes across HC, HIV and HIV-Mtb.

Group-stratified inspection of cell trajectory (**Figures 4E–G**) revealed a progressive depletion of naïve CD4^+^ T cells accompanied by a marked expansion of effector subsets, ultimately reducing the overall diversity of differentiation states. Leveraging the same UMAP embedding, we next projected module scores for four curated gene signatures—Cell-cycle, Memory, Activation and Exhaustion and applied them to the CD4^+^ subsets (**Figures 5A–E**). Consistent with the pseudotime findings, advancing disease was characterized by a stepwise increase in proliferative (Cell-cycle) and effector/activation programs, a concomitant decline in memory-associated transcriptional activity and a pronounced rise in exhaustion signatures, with the HIV-Mtb group exhibiting the most extreme phenotype. The pseudotime analysis also yielded 12 shared genes (**Supplementary Figure S7**), but their patterns did not clearly map onto stages of disease progression.

### 3.4 Network topology reconfiguration via multiple signaling pathways associated with T cells reconfiguration in HIV-Mtb co-infection

**Figure 6:**
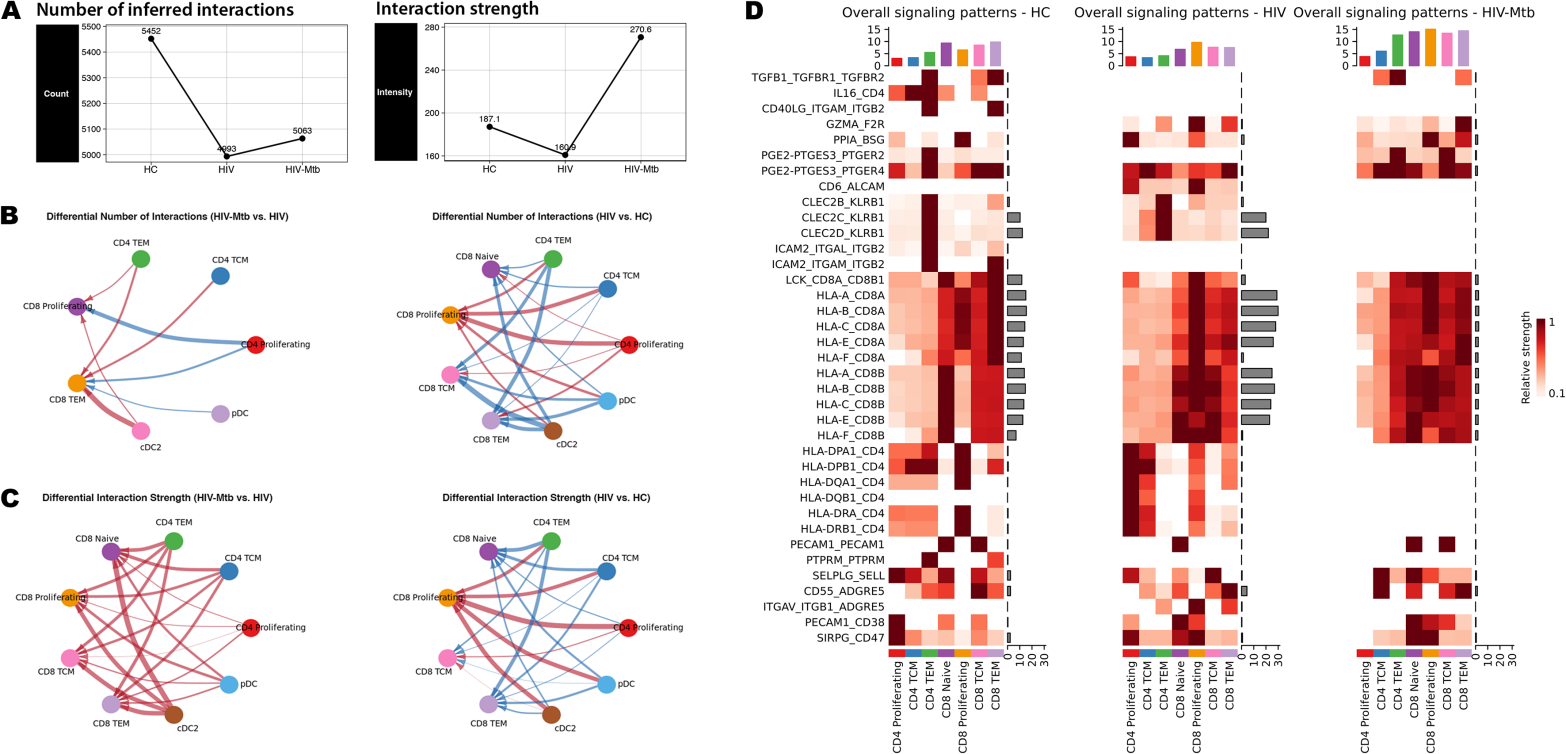
Cell-cell communication landscapes of T cell subsets. **(A)** Comparison of the total number of inferred ligand–receptor interactions (left) and the aggregate interaction strength (right) for each group. **(B)** Chord diagrams showing the number of interactions from the CD4^+^ T and dendritic cell **(DC)** subpopulations to CD8^+^ T cells; Left: the comparison between the HIV-Mtb group and the HIV group; Right: comparison between the HIV group and the HC group; Red line indicates that the cell pairs in the first group show an increase in interaction number or signaling strength; Blue line indicates a decrease; The thickness of the line reflects the magnitude of change. **(C)** Chord diagrams for the interaction strength from the CD4^+^ T and DC subpopulations to CD8^+^ T cells; Left: comparing the HIV-Mtb group with the HIV group; Right: comparing HIV group with HC group. **(D)** Heatmaps of overall signaling patterns in HC, HIV and HIV-Mtb. Rows correspond to ligand–receptor pairs with significant alterations. For each panel: Left bar plots show the total outgoing signal strength contributed by each sender cell subset. Top bar plots summarize the total signal strength for each signaling pathway. Heatmap color intensity represents relative signaling strength (darker = higher).

To further characterize signaling changes accompanying T-cell subset activation and exhaustion during HIV-Mtb co-infection and to determine whether this phenomenon is unique to T cells, we assessed global intercellular communication. As shown in **Figure 6A**, the inferred communication frequency was reduced in both HIV and HIV-Mtb groups relative to HC, with only a slight rebound in the co‐infection group. In contrast, overall interaction strength was markedly increased in HIV-Mtb, and T cell populations exhibited the most pronounced elevation in interaction strength, especially under co‐infection (**Supplementary Figure S8**). This pattern was visually confirmed by the Chord Diagrams (**Figures 6B, C**).

Next, we interrogated the ligand-receptor pairs driving T-cell subsets interactions by extracting all candidate interactions from CellChat database across the three groups and taking their union. We then focused on the pairs highlighted in **Figure 6D** as the top-ranked correlates of altered signaling. The most significant alteration in HIV-Mtb co-infection was the near complete loss of MHC class II interactions, replaced by MHC class I engagement, particularly evident in CD4^+^ TEM cells. A similar switch was seen for CLEC2B/C/D-KLRB1 signaling. Co-infection was accompanied by substantial upregulation of inhibitory pathways, including prostaglandin E2 biosynthesis (PGE2-PTGES3-PTGER2/4), cyclophilin A signaling (PPIA-BSG), and PECAM1-mediated interactions, all known to suppress T-cell activation. In parallel, ADGRE5-ALCAM-related co-stimulatory and adhesion interactions showed pair-specific dynamics: CD6-ALCAM and ITGAV-ITGB1-ADGRE5 were most prominent in the HIV-mono group and diminished with Mtb co-infection, whereas CD55-ADGRE5 did not display a comparable monotonic trend. Notably, TGF-β signaling (TGFB1-TGFBR1-TGFBR2) was extinguished during HIV-mono infection yet reappeared in co-infection, whereas IL16-CD4 and CD40LG-(ITGAM/ITGB2) interactions disappeared with HIV-mono and did not recover, underscoring stage-specific rewiring of T-cell communication networks.

As described, the CD4^+^ TEM subset exhibited significantly increased signaling intensity specifically in the HIV-Mtb group (**Figure 6D**, **Supplementary Figure S7**). Further analysis of the communication patterns of the CD4^+^ subpopulations (**Supplementary Figure S8C**) showed that CD4^+^ TEM cells exhibited strong predicted engagement with MHC I–mediated interactions in the HIV-Mtb group. Specifically, this subset in the HIV-Mtb group exhibited the lowest cellular frequency but the highest signaling intensity. We visualized the CD4^+^ TEM subset and found that this subset expressed both CD4 and CD8 in the HC and HIV groups, but in the HIV-Mtb group, CD4 expression was undetectable (0%) (**Supplementary Figure S9A**), consistent with predominant MHC I–restricted engagement. Additionally, this subset maintained stable expression of TCRα and TCRβ chain constant region genes (TRAC, TRBC1/2) (**Supplementary Figure S9B** Right), suggesting that they may be αβ T cells.

## 4 Discussion

HIV-Mtb co-infection demonstrates greater mechanistic complexity and hyperactivation than HIV-mono infection. Beyond the CD8^+^ T cells hyperactivation described in the results, our integrated enrichment and pseudotime trajectory analyses both revealed that multiple CD4^+^ T subpopulations initiate apoptosis and undergo reserve depletion in chronic HIV infection. These processes became even more pronounced during co-infection, paradoxically coinciding with markedly heightened inflammation. Accordingly, this discussion will focus on the paradoxical amplification of these responses and the consequences of CD4^+^ T-cell reserve depletion.

On this foundation, the study revealed that Th1 cells not only became the main anti-tuberculosis effector cells in HIV-infected individuals during co-infection but also exhibited an increasingly complex functional profile. At the pathway level, despite a dramatic expansion of Th1 cells in co-infection, integrated analyses revealed functional paradoxes driving disease progression, as the enrichment of Th17 differentiation and TNF signaling in Class1 indicates immune activation with a risk of uncontrolled inflammation and tissue damage (25, 26). By contrast, the enrichment of HIV-1 infection and PD-1/PD-L1 pathways in Class2 suggests increased intracellular viral activity and functional exhaustion. Notably, bacterial infection pathways were unique to Class2. We interpret this selective enrichment as Mtb-driven activation of shared host-response modules, including cytoskeletal remodeling and pattern-recognition/apoptotic signaling (e.g., STING1, TRADD, CASP8), that are commonly annotated under bacterial KEGG terms. In contrast, Class1 was dominated by viral-infection annotations and antigen presentation (e.g., HLA-DRB1/DQB1/C, CD74, CTLA4, GZMB, PRF1), consistent with HIV-associated immune activation. Thus, co-infection shifts the balance of infection-related programs rather than abolishing antiviral pathways, as viral terms remain detectable in Class2, whereas bacterial pathways are confined to Class2. At the molecular level, Class1 also involved TB pathway–related regulators such as HLA-DPA1 and TGFB1, which are linked to impaired antigen presentation and the induction of anti-inflammatory cytokines (27–29). In Class2, molecules like STING1 were enriched in the HIV-1 infection pathway, indicating that co-infection upregulated STING1 to promote the expression of antiviral genes (30). Additionally, we uncovered a complex yet paradoxical expression pattern within Th1 immune responses. IFN-γ secreted by Th1 is critically involved in both antiviral and anti-tuberculosis responses, and it is known that STAT1 is a core mediator of interferon signaling (31), while STAT3 can be upregulated by HIV infection and activated by IFN-γ, promoting the secretion of IL-6 and IL-10, which drive chronic inflammation (32, 33). Therefore, we analyzed the differential expression of STAT family genes (**Supplementary Table S2**) and found that HIV-mono infection leads to the upregulation of STAT3 and STAT4, whereas co-infection results in the upregulation of STAT1 and STAT2. The former is consistent with previous research suggesting that HIV vif can suppresses type I interferon pathways to achieve immune evasion (34), while the latter indicates that Mtb co-infection may also activate immune responses detrimental to HIV. Nonetheless, persistent STAT3 activation and further upregulation of STAT4 — a key regulator of Th1 differentiation (35) — could exacerbate chronic inflammation and tissue damage in co-infection. We believe that elucidating the complex behavior of Th1 cells is pivotal to effectively controlling inflammation during co-infection.

Integrating cellchat analysis, we found that co-infection induced significant plasticity in CD4^+^ T cell differentiation, shifting the terminal state from conventional Th1 dominance toward a Th1/Th17 hybrid phenotype. This transition, mapped through pseudotime trajectory analysis (**Figure 4G**), likely reflects the influence of TB-associated inflammatory mediators (e.g., IL-1β, IL-12), which drive Th17 cells to acquire Th1-like functions (36). Conversely, Th17 subset was largely consistent with previous studies, showing earlier exhaustion in HIV infection (37, 38), and its functionality was severely compromised (**Supplementary Figure S6**), consistent with the early loss of IL-17–producing CD4^+^ T cells in HIV infection (39) and their sustained exhaustion during co-infection (40). Notably, Mtb-specific Th17 cells were markedly depleted in co-infected individuals, with frequencies remaining low despite ART or TB therapy, indicating an irreversible deficit (40, 41). This Th1-to-Th1/Th17 plasticity represents a novel differentiation axis uniquely amplified in co-infection.

Observations across the remaining cell subsets were aligned with previous studies. For CD4^+^ naive T cells, enrichment analysis revealed inflammatory dysregulation largely consistent with Th1. Moreover, we found that co-infection induced downregulation of the NF-κB pathway. This is abnormal for the canonical NF-κB pathway, which is normally associated with the secretion of pro-inflammatory factors like TNF-α and IL-6 and inflammasome formation (42), and it can be persistently activated by HIV via Tat protein or Toll-like receptors (43, 44). Whether this phenomenon is prevalent across co-infected patients still need further investigation. Turning to TFH cells, we observed a divergent functional profile. In HIV-mono, despite numerical expansion, their function was compromised through HIV tat-mediated pathways and ubiquitin ligase activity, suppressing IL-21 expression (45–47). During co-infection, TFH cells displayed enrichment in p53 signaling, TLR, and RAGE pathways—indicating chronic inflammation, apoptosis, and exhaustion. Uniquely, ribosome-related pathways were partly enriched in Class2 in co-infection, suggesting pathogen exploitation of cellular resources to support HIV replication or Mtb metabolic demands.

With evidence of paradoxical activation, we also found the pathways that enforce T-cell dysfunction and exhaustion during co-infection: the PD-1/PD-L1 pathway was enriched across all subpopulations. Specifically, Th1, Th17, and TFH cells upregulated inhibitory signals (e.g., PDCD1, CD274, CTAL4), which encode T-cell suppression markers (48). We also found that co-infection reconfigured intercellular signaling networks toward potent immunosuppression. Key inhibitory mediators were significantly upregulated, including: (i) Prostaglandin E2 synthesis (PGE2–PTGES3–PTGER2/4), which directly suppresses TCR signaling and T cell proliferation (49); (ii) Cyclophilin A–CD147 interactions, inhibiting calcineurin/NFAT-driven T cell activation and IL-2 production while disrupting ERK1/2-mediated chemotactic migration (50); and (iii) Homotypic and heterotypic PECAM1 (CD31) pathways, facilitating TGF-β–mediated suppression of T cell activity (51). In result 3.4, CLEC2B/C/D-KLRB1 signaling was significantly present in both the HC and mono-infection groups but completely disappeared in co-infection group. By consulting the database, we found that the CLEC2D-KLRB1 interaction plays a crucial role in T cell immune regulation, promoting T cell proliferation and IFN-γ secretion (52). Previous studies have shown that HIV infection can lead to a reduction in CD161^+^ T cells (53), and co-infection with MTB may further exacerbate this depletion or functional exhaustion. Therefore, we speculate that the absence of the CLEC2D-KLRB1 interaction in HIV-MTB co-infection mainly reflects a defect at the immune receptor end, accompanied by the disruption of key immune interactions. As for the CLEC2B-KLRB1 and CLEC2C-KLRB1 interactions, there is currently a lack of direct experimental evidence to support them, and their presence is more derived from the database’s inference based on homologous family molecules. Additionally, the selective weakening of the ADGRE5-ALCAM co-stimulatory axis, such as the downregulation of CD6-ALCAM, further restricts the complete activation of T cells. This collective upregulation signifies a pathological shift from broad network attenuation to targeted amplification of exhaustion-promoting signals, driving rapid functional impairment upon T cell activation. Therefore, we propose that despite the presence of some seemingly paradoxical immune responses, co-infection overall demonstrates synergistic pathogen interactions that drive host immune exhaustion.

Notably, within CD4^+^ effector memory T cells (TEM), we identified a distinct subset exhibiting unconventional activation via MHC I rather than MHC II pathways. These cells, likely αβ T cells, demonstrated a phenotypic shift toward CD8-like differentiation (**Supplementary Figure S9**) and showed dramatically enhanced MHC I-mediated activation signals in co-infection (**Figure 6**, **Supplementary Figure S8**). This aligns with studies linking peripheral αβ T cell depletion in active TB to compromised immunity (54), and evidence that Mtb-responsive αβ T cells detected by activation markers (CD69/CD154/CD137) dominate early immune responses (55). The aberrant MHC I engagement on CD4^+^ TEM cells underscore a co-infection-specific mechanism of T cell dysregulation. Collectively, these data suggested this αβ T subset may play a role in the immune response during HIV-Mtb co-infection, though its potential host-damage effects still require further investigation.

We acknowledge that this study has several limitations that should be taken into consideration when interpreting the results. Firstly, the relatively small sample size in our single-cell sequencing experiments may introduce sampling bias, affecting the representativeness and reproducibility of the results. Secondly, functional validation of the aberrant CD4^+^ TEM subset was not performed. Although we identified its non-canonical MHC-I-restricted activation and CD4 surface loss, these findings remain inferential. Moreover, the roles of PD-1, STATs, and TNF identified in our analysis represent exploratory hypotheses that warrant additional validation in future studies. Finally, our New Method was only applied to the KEGG database for enrichment analysis; future work should expand this approach to include other mainstream databases such as GO, WikiPathway, and REACTOME.

Our study demonstrates that HIV-Mtb co-infection profoundly reshapes CD4^+^ T cells immunity by driving exhaustion and functional dysregulation. CD8^+^ T cells and MHC I–dominated responses appear overreactive and may contribute to collateral tissue damage. Integrated analyses indicate increased MHC I–mediated signaling (e.g., a non-canonical CD4^+^ TEM axis) and a rebalancing of infection-related programs. The intersection of immune exhaustion and dysregulated inflammation exposes actionable vulnerabilities. Targeting pathways such as PD-1/PD-L1, TNF signaling, STAT family modulation, together with rewired cell–cell interactions characterized by increased inhibitory checkpoints (PGE2–PTGES3–PTGER2/4, PPIA–BSG, PECAM1) and reduced co-stimulatory signals (CD6–ALCAM, CLEC2B/C/D–KLRB1), may provide avenues for precision immunomodulation in co-infected individuals, pending further validation. These findings highlight that in HIV-Mtb co-infection, pathways are not “simultaneously suppressed or activated” but exhibit a finely tuned, spatiotemporal interplay.

## Data Availability

The datasets presented in this study can be found in online repositories. The names of the repository/repositories and accession number(s) can be found in the article/**Supplementary Material**. The raw data have been deposited in GEO under accession number GSE293960 (https://www.ncbi.nlm.nih.gov/geo/query/acc.cgi?acc=GSE293960). Source code related to this study is available at our GitHub repository: https://github.com/tony27786/ThreeGroupQuadDiff.
