# Supplementary material for: Single‐cell transcriptomics reveals pathogen interactions and T cell reprogramming in HIV and Mycobacterium tuberculosis co‐infection
Source: Front Immunol. 2025 Nov 28;16:1680538. doi: 10.3389/fimmu.2025.1680538 (PMC12698600; doi:10.3389/fimmu.2025.1680538)
Supplement: Supplementary file 1 [file Table1.docx]

Supplementary Material

# Data and code availability

All the datasets used in this paper are publicly available and can be downloaded from https://www.ncbi.nlm.nih.gov/geo/query/acc.cgi?acc=GSE293960. Our novel method and its accompanying instructions are available at https://github.com/tony27786/ThreeGroupQuadDiff.

# Supplementary Figures


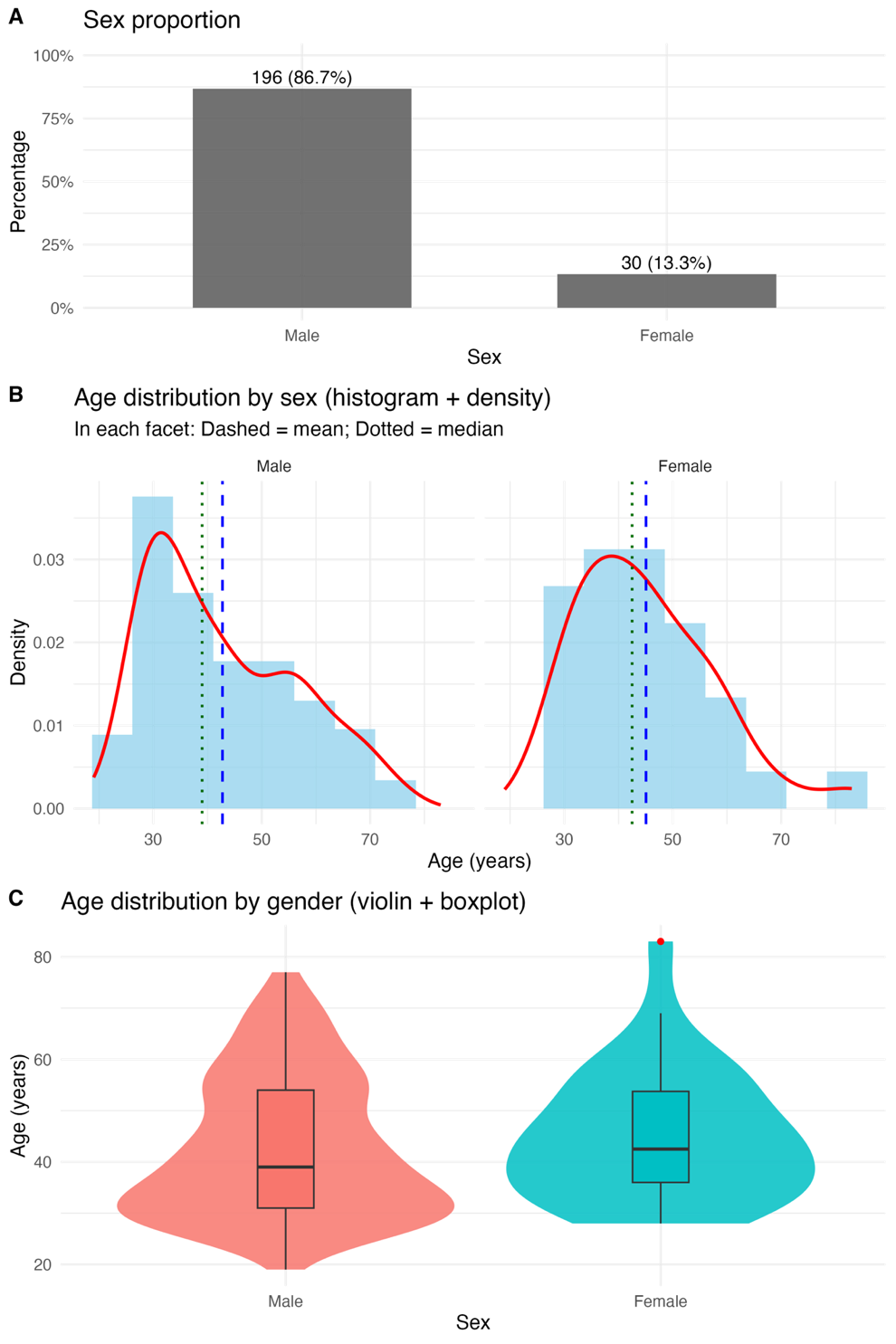


**Supplementary Figure S1. Sex and age distribution in our local cohort.** (A) Sex composition of the local population initially assessed for enrollment. (B) Age distribution histogram with density curves stratified by sex (dashed line = mean; dotted line = median). (C) Violin and boxplot showing age distribution by sex.


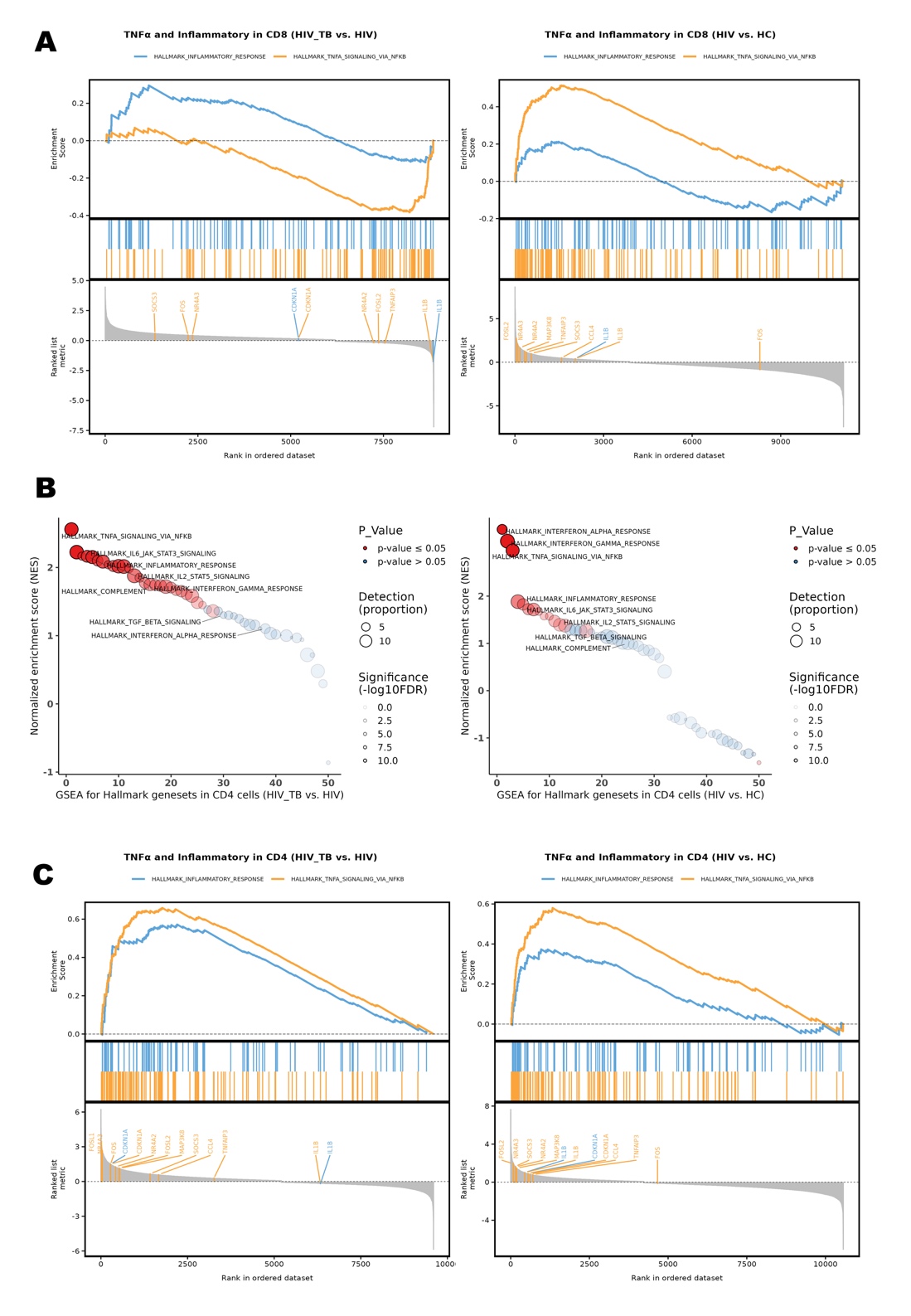


**Supplementary Figure S2. GSEA plots for CD4^+^ and CD8^+^ T cells**. (A) GSEA pathway plot of TNFα and Inflammatory for CD8^+^ T cells (Left: HIV-Mtb vs. HIV; Right: HIV vs. HC). (B) GSEA analysis of 50 Hallmark pathways in CD4^+^ T cells across three groups (Left: HIV-Mtb group vs. HIV group; Right: HIV group vs. HC group). (C) GSEA pathway plot of TNFα and Inflammatory for CD4^+^ T cells (Left: HIV-Mtb vs. HIV; Right: HIV vs. HC).

**
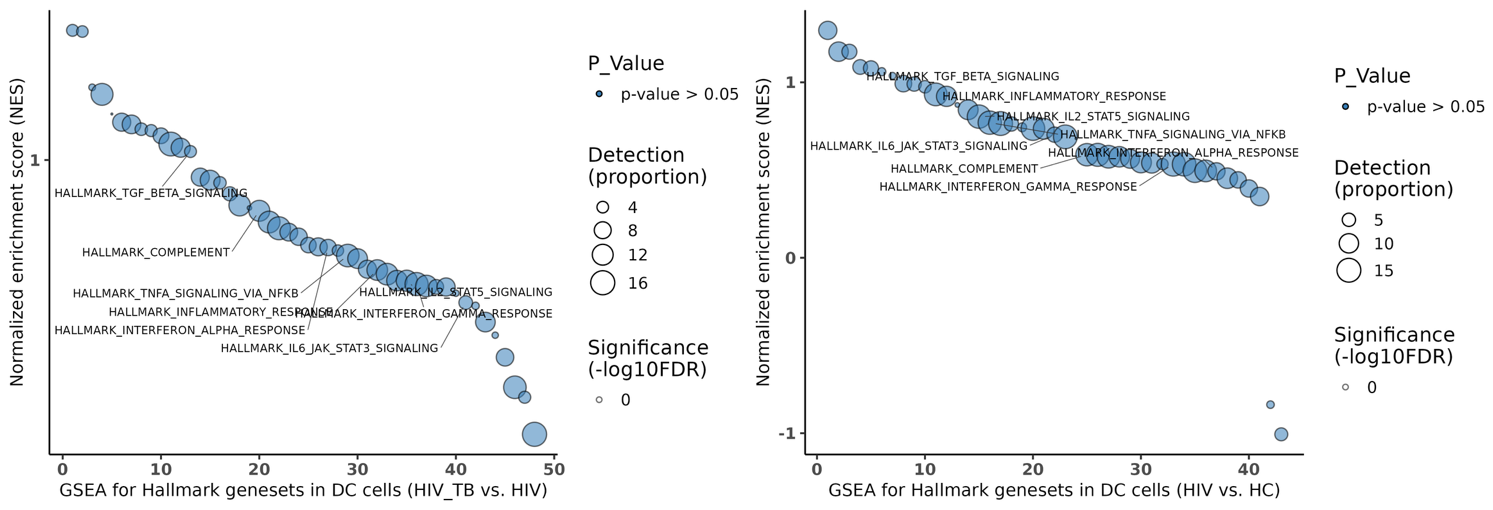
**

**Supplementary Figure S3. Hallmark GSEA screening for dendritic cells.** Left: HIV-Mtb group vs. HIV group; Right: HIV group vs. HC group.

**
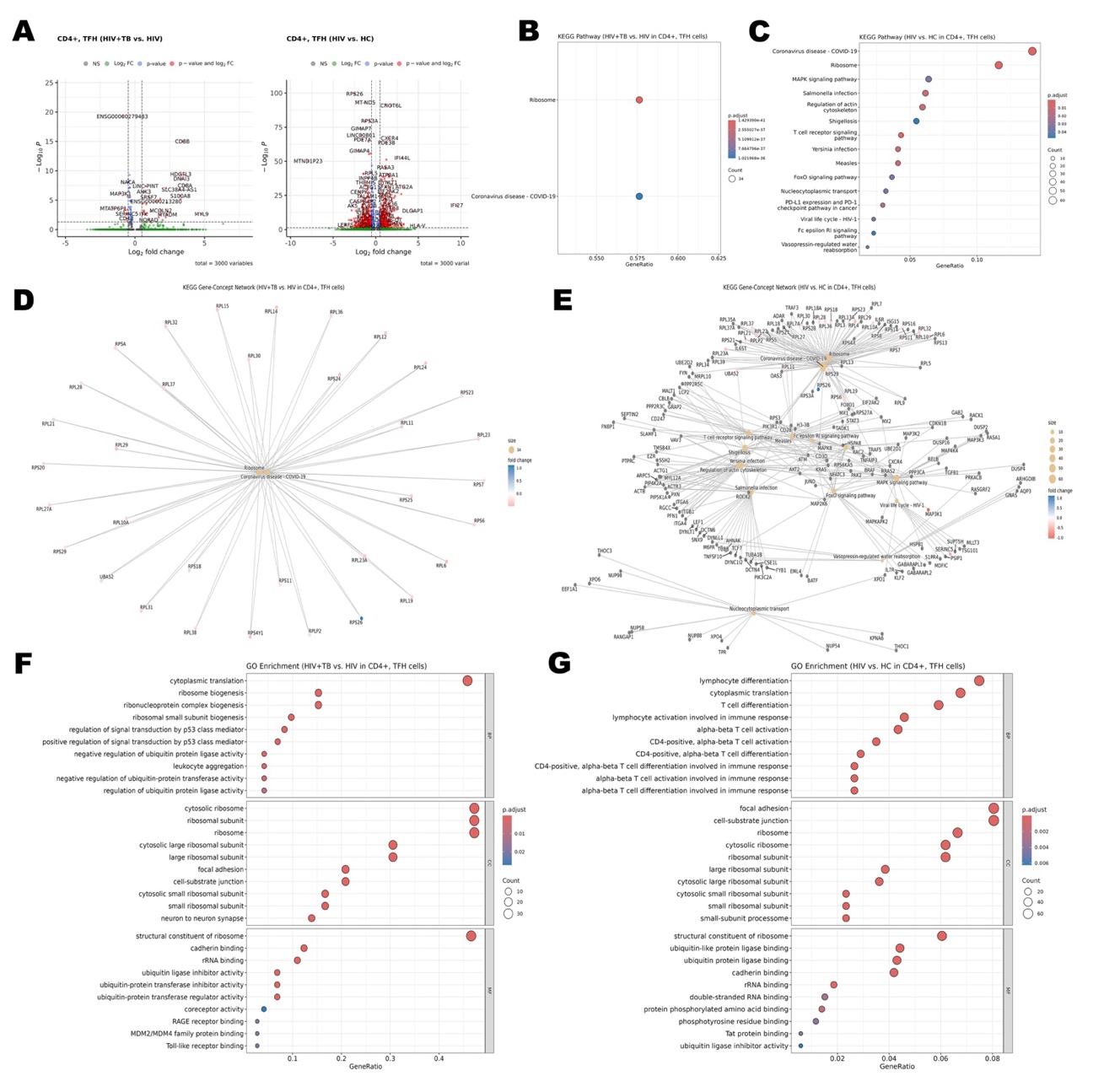
**

Supplementary Figure S4. Differential expression pairwise comparison results for the CD4^+^ TFH cell subpopulation among three groups. (A) Volcano plots for pairwise comparisons: Left panel, HIV-Mtb vs. HIV; Right panel, HIV vs. HC. (B) Dotplot of KEGG (Kyoto Encyclopedia of Genes and Genomes) enrichment analysis comparing HIV-Mtb to HIV. (C) Dotplot of KEGG enrichment analysis comparing HIV to HC. (D) Gene-Concept Network derived from KEGG enrichment results for HIV-Mtb vs. HIV. (E) Gene-Concept Network derived from KEGG enrichment results for HIV vs. HC. (F) Dotplot of GO (Gene Ontology) term enrichment analysis results for HIV-Mtb vs. HIV; the top, middle, and bottom panels represent BP (Biological Process), CC (Cellular Component), and MF (Molecular Function), respectively. (G) Dotplot of GO term enrichment analysis results for HIV vs. HC.


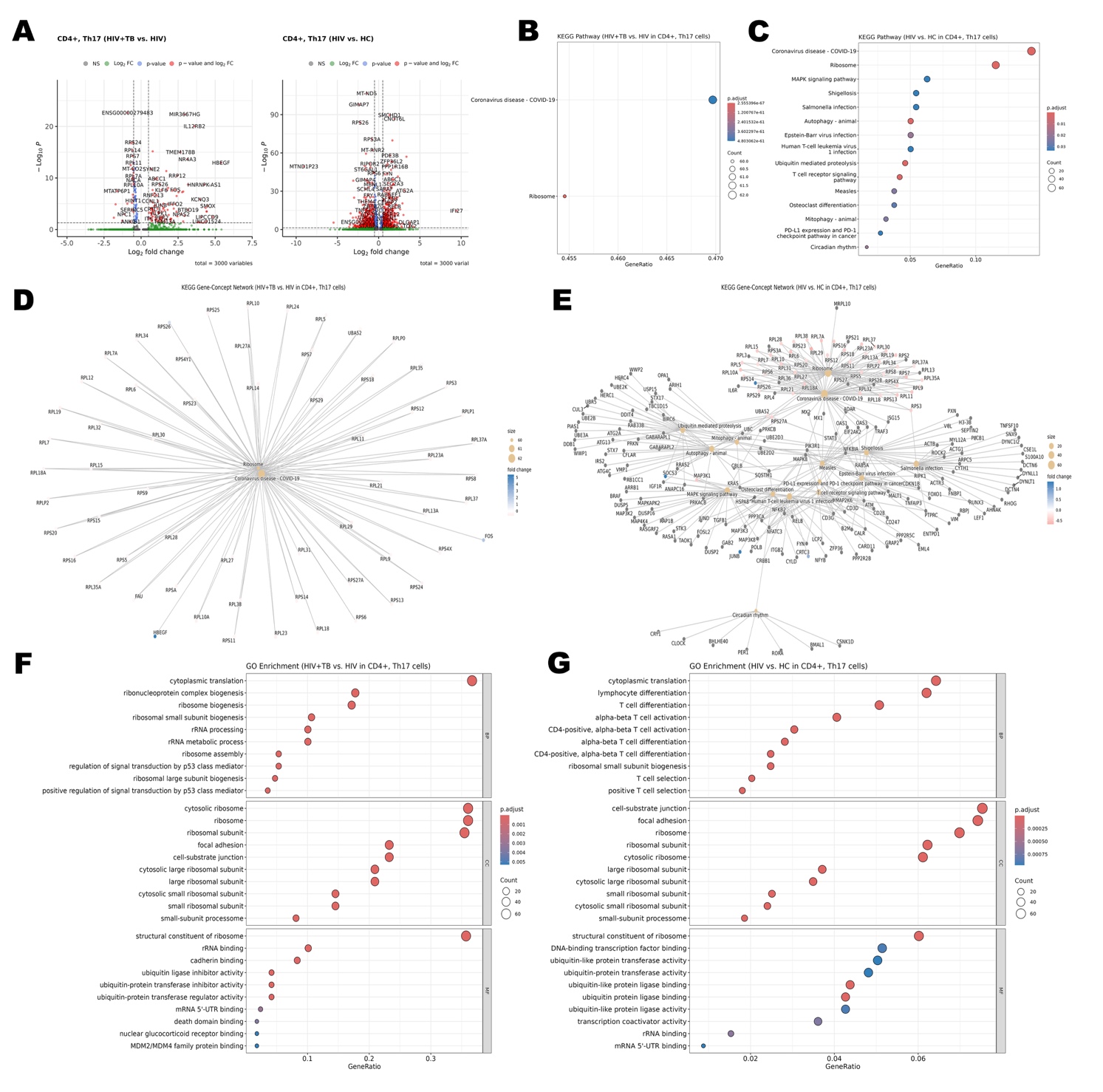


Supplementary Figure S5. Differential expression pairwise comparison results for the CD4^+^ Th17 cell subpopulation among three groups. (A) Volcano plots for pairwise comparisons: Left panel, HIV-Mtb vs. HIV; Right panel, HIV vs. HC. (B) Dotplot of KEGG (Kyoto Encyclopedia of Genes and Genomes) enrichment analysis comparing HIV-Mtb to HIV. (C) Dotplot of KEGG enrichment analysis comparing HIV to HC. (D) Gene-Concept Network derived from KEGG enrichment results for HIV-Mtb vs. HIV. (E) Gene-Concept Network derived from KEGG enrichment results for HIV vs. HC. (F) Dotplot of GO (Gene Ontology) term enrichment analysis results for HIV-Mtb vs. HIV; the top, middle, and bottom panels represent BP (Biological Process), CC (Cellular Component), and MF (Molecular Function), respectively. (G) Dotplot of GO term enrichment analysis results for HIV vs. HC.


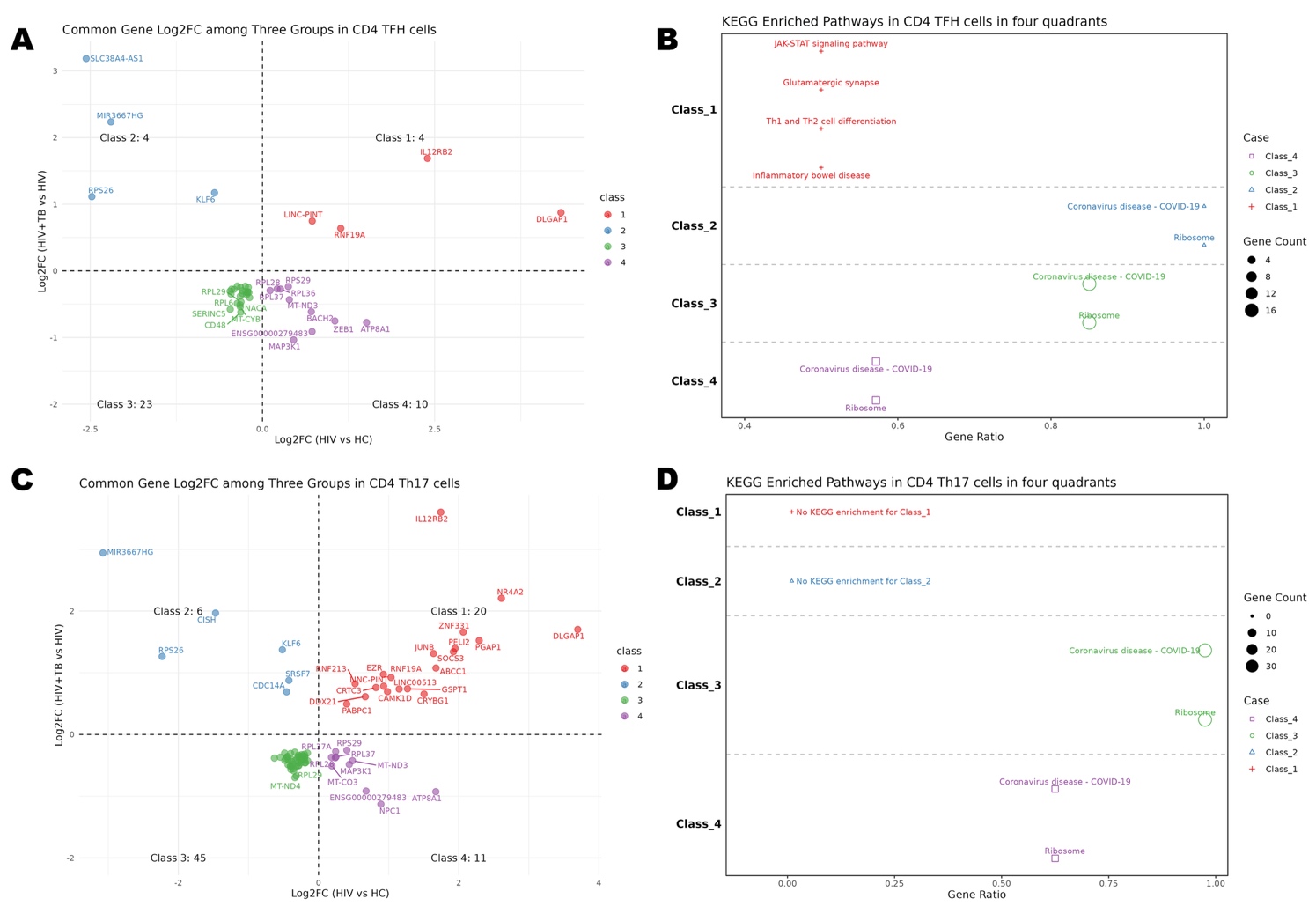


Supplementary Figure S6. Group-based time-series analysis of CD4^+^ T cell subsets using the novel method. (A) Differential Expression Four-Quadrant Plot of CD4^+^ TFH Cells; the X-axis represents the comparison between the HIV group and the HC group, and the Y-axis represents the comparison between the HIV-Mtb group and the HIV group. (B) KEGG Enrichment Analysis Results of the Molecules Corresponding to the Quadrants of CD4^+^ TFH Cells. (C) Differential Expression Four-Quadrant Plot of CD4^+^ Th17 Cells. (D) KEGG Enrichment Analysis Results of the Molecules Corresponding to the Quadrants of CD4^+^ Th17 Cells.


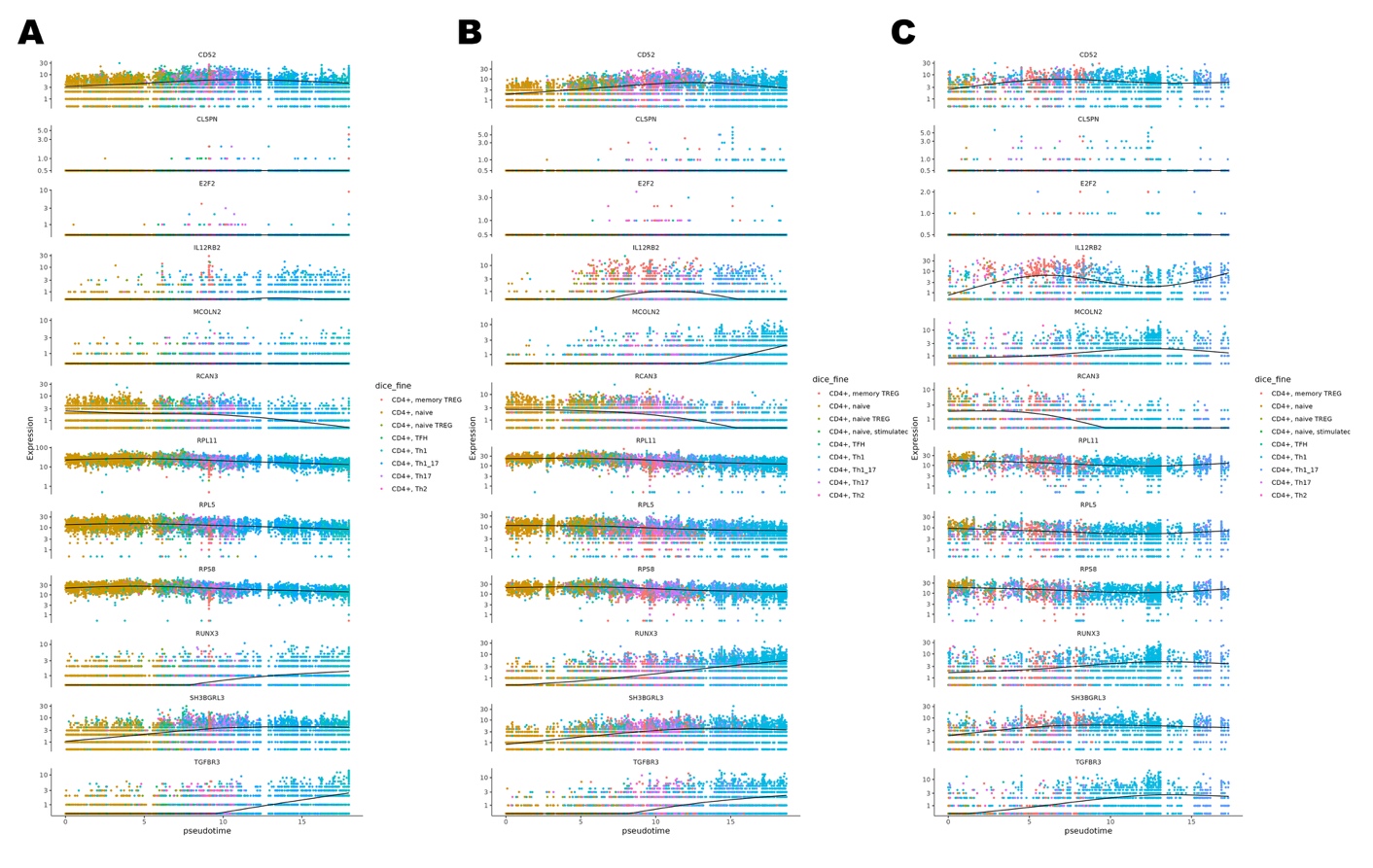


Supplementary Figure S7. Pseudotime-Gene curve of selected gene by Moran’s I statistics. (A) Gene Expression Curve of Selected Molecules in the HC Group, displaying the expression of individual genes along pseudotime. (B) Gene Expression Curve of Selected Molecules in the HIV Group, displaying the expression of individual genes along pseudotime. (C) Gene Expression Curve of Selected Molecules in the HIV-Mtb Group, displaying the expression of individual genes along pseudotime.


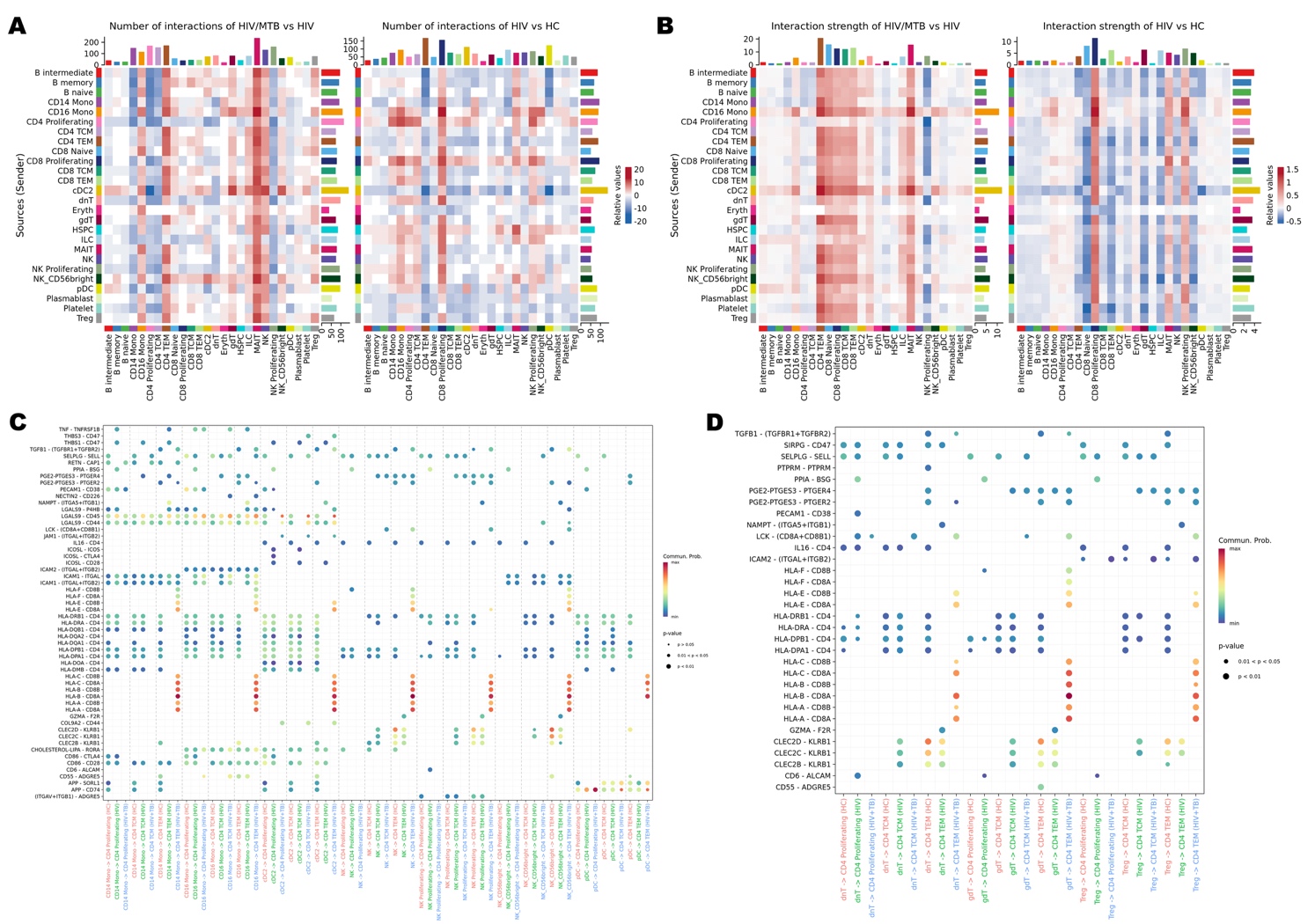


Supplementary Figure S8. CellChat analysis of interactions among different cell populations. (A) Heatmap showing differential number of interactions among different cell populations (Left: HIV-Mtb vs. HIV; Right: HIV vs. HC). (B) Heatmap showing differential interaction strength among different cell populations (Left: HIV-Mtb vs. HIV; Right: HIV vs. HC). (C) Cell interaction plot of of APCs and NK cell subpopulations with CD4^+^ T cell subpopulations. Y-axis represents the “ligand-receptor pairs”, and the X-axis represents the interacting cell subsets; circle size corresponds to P-value; the color—ranging from blue to red—reflects the communication probability, with red indicating higher probabilities. (D) Cell interaction plot of non‑classical CD4^+^ T cell subpopulations with classical CD4^+^ T cell subpopulations.


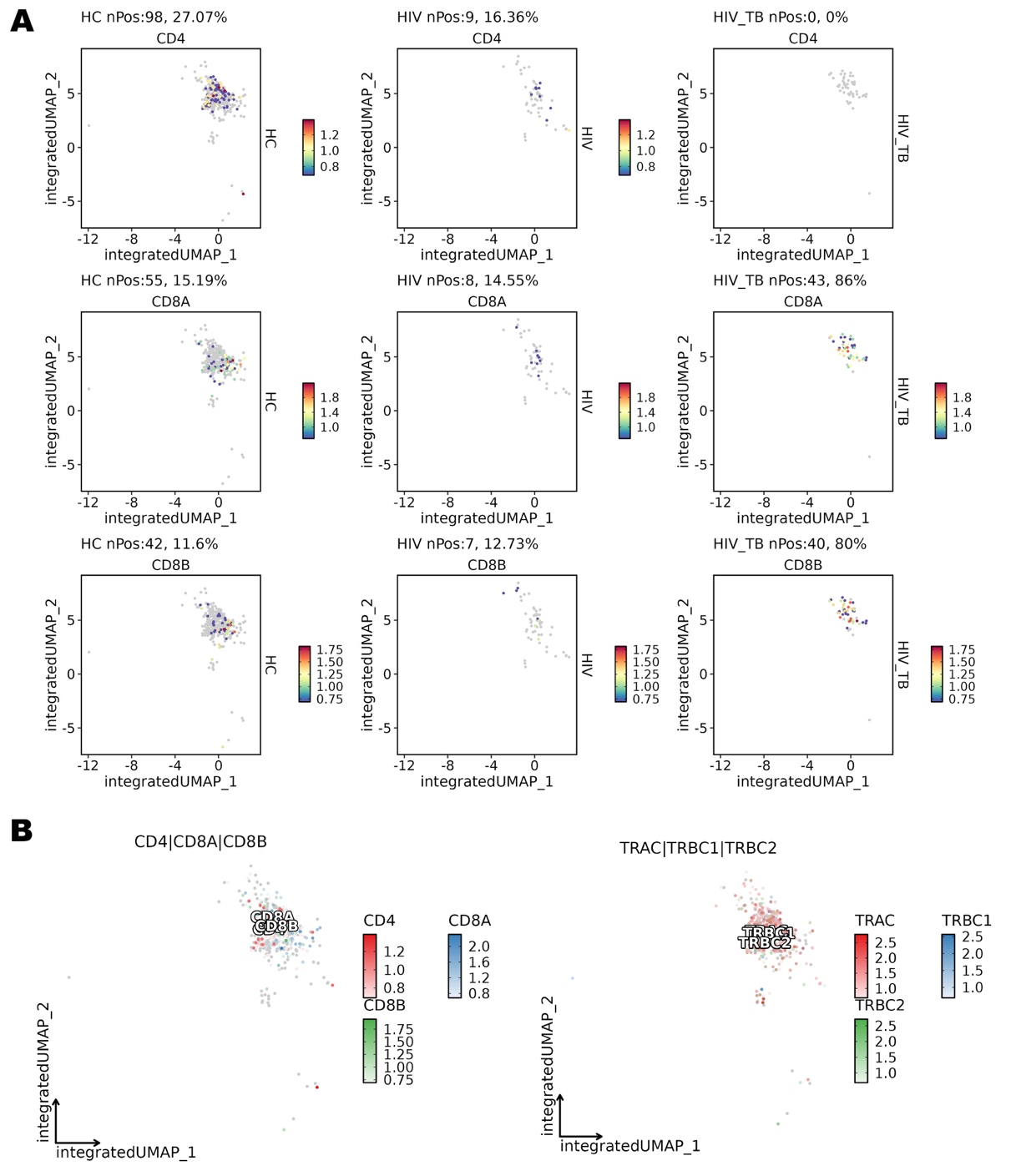


Supplementary Figure S9: Further analysis of the increased strength in the CD4^+^ TEM subset. (A) UMAP plots displaying the expression levels of CD4^+^ and CD8^+^ T cells gene signatures in the isolated CD4^+^ TEM subset from the three groups, arranged from left to right as Healthy Control, single HIV infection, and HIV-Mtb co-infection. “nPos” indicates the percentage of cells positive for the respective gene out of the total cell count. (B) Left panel shows the merged annotation plot distinguishing the CD4^+^ and CD8^+^ T cells gene signature, revealing the convergence of similar cell expression patterns; Right panel displays the merged annotation plot of the gene signature encoding the TCR α/β chain constant region.

# Supplementary Tables

Supplementary Table 1. Welch’s t-test on the relative frequencies of T cell subsets

| **CellType** | **Group1** | **Group2** | ***P*** | **p.signif** |
| --- | --- | --- | --- | --- |
| CD4+, naive | HC | HIV | 0.1170 | ns |
| CD4+, naive | HC | HIV-Mtb | 0.0124 | * |
| CD4+, naive | HIV | HIV-Mtb | 0.1400 | ns |
| CD4+, naive, stimulated | HC | HIV | 0.5720 | ns |
| CD4+, naive, stimulated | HC | HIV-Mtb | 0.3260 | ns |
| CD4+, naive, stimulated | HIV | HIV-Mtb | 0.6540 | ns |
| CD4+, naive TREG | HC | HIV | 0.1230 | ns |
| CD4+, naive TREG | HC | HIV-Mtb | 0.0861 | ns |
| CD4+, naive TREG | HIV | HIV-Mtb | 0.0085 | ** |
| CD4+, memory TREG | HC | HIV | 0.0563 | ns |
| CD4+, memory TREG | HC | HIV-Mtb | 0.0634 | ns |
| CD4+, memory TREG | HIV | HIV-Mtb | 0.9340 | ns |
| CD4+, Th1 | HC | HIV | 0.0909 | ns |
| CD4+, Th1 | HC | HIV-Mtb | 0.0138 | * |
| CD4+, Th1 | HIV | HIV-Mtb | 0.2030 | ns |
| CD4+, Th1_17 | HC | HIV | 0.8410 | ns |
| CD4+, Th1_17 | HC | HIV-Mtb | 0.6040 | ns |
| CD4+, Th1_17 | HIV | HIV-Mtb | 0.7470 | ns |
| CD4+, Th17 | HC | HIV | 0.0068 | ** |
| CD4+, Th17 | HC | HIV-Mtb | 0.6070 | ns |
| CD4+, Th17 | HIV | HIV-Mtb | 0.0129 | * |
| CD4+, Th2 | HC | HIV | 0.6310 | ns |
| CD4+, Th2 | HC | HIV-Mtb | 0.0310 | * |
| CD4+, Th2 | HIV | HIV-Mtb | 0.0612 | ns |
| CD4+, TFH | HC | HIV | 0.0058 | ** |
| CD4+, TFH | HC | HIV-Mtb | 0.5590 | ns |
| CD4+, TFH | HIV | HIV-Mtb | 0.0030 | ** |
| CD8+, naive | HC | HIV | 0.1260 | ns |
| CD8+, naive | HC | HIV-Mtb | 0.4430 | ns |
| CD8+, naive | HIV | HIV-Mtb | 0.3760 | ns |
| CD8+, naive, stimulated | HC | HIV | 0.2390 | ns |
| CD8+, naive, stimulated | HC | HIV-Mtb | 0.8470 | ns |
| CD8+, naive, stimulated | HIV | HIV-Mtb | 0.3110 | ns |

ns: not significant; *: *P*<0.05; **: *P*<0.01

Supplementary Table 2. Differential expression analysis of the STAT family in CD4^+^ Th1

| **Gene** | **Group1** | **avg_log2FC** | ***P_adj*** | **Group2** | **avg_log2FC** | ***P_adj*** |
| --- | --- | --- | --- | --- | --- | --- |
| STAT1 | HIV vs. HC | - | - | HIV-Mtb vs. HIV | 0.911405553 | ＜0.001 |
| STAT2 | HIV vs. HC | 0.51097625 | 1 | HIV-Mtb vs. HIV | 0.716366448 | ＜0.001 |
| STAT3 | HIV vs. HC | 1.011473678 | ＜0.001 | HIV-Mtb vs. HIV | 0.191915424 | 1 |
| STAT4 | HIV vs. HC | 0.248794982 | ＜0.001 | HIV-Mtb vs. HIV | 0.27231508 | ＜0.001 |
| STAT5A | HIV vs. HC | 2.01903137 | ＜0.001 | HIV-Mtb vs. HIV | -0.230826654 | 1 |
| STAT5B | HIV vs. HC | -0.11714895 | 1 | HIV-Mtb vs. HIV | - | - |
| STAT6 | HIV vs. HC | -0.49826589 | 0.0808 | HIV-Mtb vs. HIV | 0.312708367 | 1 |

*P*‑values were adjusted for multiple testing to control the false discovery rate (FDR) using the Benjamini–Hochberg procedure.
